# Methodological aspects of EEG and body dynamics measurements during motion

**DOI:** 10.3389/fnhum.2014.00156

**Published:** 2014-03-24

**Authors:** Pedro M. R. Reis, Felix Hebenstreit, Florian Gabsteiger, Vinzenz von Tscharner, Matthias Lochmann

**Affiliations:** ^1^Department of Sports and Exercise Medicine, Institute of Sport Science and Sport, Friedrich-Alexander-University Erlangen-NurembergErlangen, Germany; ^2^Digital Sports Group, Pattern Recognition Lab, Department of Computer Science, Friedrich-Alexander-University Erlangen-NurembergErlangen, Germany; ^3^Human Performance Laboratory, Faculty of Kinesiology, University of CalgaryCalgary, AB, Canada

**Keywords:** electroencephalography, methodology, hardware and software, movement and exercise, artifacts reduction, electrodes digitalization

## Abstract

EEG involves the recording, analysis, and interpretation of voltages recorded on the human scalp which originate from brain gray matter. EEG is one of the most popular methods of studying and understanding the processes that underlie behavior. This is so, because EEG is relatively cheap, easy to wear, light weight and has high temporal resolution. In terms of behavior, this encompasses actions, such as movements that are performed in response to the environment. However, there are methodological difficulties which can occur when recording EEG during movement such as movement artifacts. Thus, most studies about the human brain have examined activations during static conditions. This article attempts to compile and describe relevant methodological solutions that emerged in order to measure body and brain dynamics during motion. These descriptions cover suggestions on how to avoid and reduce motion artifacts, hardware, software and techniques for synchronously recording EEG, EMG, kinematics, kinetics, and eye movements during motion. Additionally, we present various recording systems, EEG electrodes, caps and methods for determinating real/custom electrode positions. In the end we will conclude that it is possible to record and analyze synchronized brain and body dynamics related to movement or exercise tasks.

## 1. Introduction

Eighty-four years passed since Hans Berger recorded the first human electroencephalogram, thus the creation of EEG (Berger, 1929; La Vaque, 1999). Methods and applications have come a long way since then. Indeed, clinicians and researchers nowadays use EEG in the management of epilepsy, monitoring of coma patients, investigation of stroke; sleep dysfunction studies, machine control, sports performance amongst others. This method is often preferred to others because it is relatively cheap, easy to wear, light weight and has a high temporal resolution. In contrast, other methods such as functional Magnetic Resonance Imaging (fMRI), have low temporal resolution, are more expensive and are impossible for study ing participants whom wear them while moving. Thus, EEG became one of the most used methods for inspecting and understanding the processes from which behavior originates.

Behavior includes all actions that beings perform in their environment, and these include motion (Vanderwolf, 2007). Makeig et al. (2009) proposed the development of methods for the investigation of brain dynamics during human motion in several dimensions and the development of wearable mobile brain/body imaging (MoBi) methodology. The authors additionally proposed the creation of analysis methods that can model the relationships between the recorded dimensions. The development of such methods will enable researchers to investigate a person's simultaneously recorded brain electric activity, muscle myoelectric activity, movements in 3D space, video, and audio recordings; thus enabling the simultaneous study of brain and body dynamics interactions during motion and behavior.

The comprehension of brain-muscle interactions is beneficial for assessing degenerative diseases, impairments of motion, designing and optimizing neuro-rehabilitation therapies, human brain machine control, human performance optimization and other applications. However, clinicians and scientists considered EEG excessively artifact prone, hence incapable of recording analyzable EEG recordings during motion. Consequently, researchers avoided using EEG recordings in movement studies and preferred indirect methods involving imagery or small limb movements to study brain activity during motion (Salenius et al., 1997; Dobkin et al., 2004; Schaal et al., 2004; Zehr and Duysens, 2004).

EEG recordings use either invasive electrodes (iEEG or ECoG) or surface electrodes (sEEG). Owning to the fact that iEEG involves direct contact with the brain, the signal to noise ratio is much higher than with surface EEG. Nevertheless, iEEG involves surgery (craniotomy) to place an electrode grid on a small portion of the brain surface. This limits the information source area that the system and experts can analyze. This can cause post-surgery problems for the subject. Further, due to ethical considerations the surgery must be indicated for the benefit of the patient. Thus it nearly always involves preparation for surgery of epileptic patients. Therefore, in general, iEEG is impractical for EEG in motion research in most populations. Hence, this paper focuses on spatial resolution and high-density motion surface EEG methodology. Consequently, we refer in this paper to sEEG simply as EEG.

We found no compilation of methodological articles or guidelines for brain and body dynamics measurements. Therefore, this paper aims to supply researchers with an overview on current hardware, software and methods for this purpose. Accordingly, we discuss issues that potentially impair the recording, analysis and recent solutions developed to address these problems. These cover suggestions of how to avoid motion artifacts, the use of custom designed accessories for EEG recording during movement, the possibility and advantages of using trans-impedance amplifiers, determination of real/custom electrode positions, EEG electrode types, the use of different EEG recording systems, artifact removal and the integration of brain, motion capture (MOCAP) and EMG recordings. As an introduction, we offer a short overview of EEG principles.

## 2. Principles of EEG

The basic functional structure of the brain is the neuron and the human brain contains about 10^11^ of them (Herculano-Houzel, 2009). Neurons are specialized cells that are able to manipulate their membrane electric potentials in order to transmit electrical signals from one to another. These electric signals, or action potentials, are rapid, instantaneous electric events. They have an amplitude of 100 mV, last 1 ms and are conducted through the axon, at a speed that varies from 1 to 100 m/s. This is the method that the brain utilizes for information exchange. This process works rather well for fast communication because of the intricate network, and amount of neurons that constitute the system (Kandel, 2000).

In an all-or-nothing chain reaction, the signal propagates throughout the network. The signal is transmitted in a wave-like movement of activation across the excitable medium of the brain which is composed of axons, synapses, dendritic membranes and ionic channels. Following an axon depolarization and the creation of an excitatory postsynaptic potential (EPSP) at neighboring dendrites, cell membrane depolarization occurs. Neurotransmitters in the excitatory synapses cause an influx of positive ions at the postsynaptic membrane. This creates a negative charge at the apical dendrites of the postsynaptic neuron. Thus a reorganization of ions ensues inside the cell. Ions move from the apical dendrite to the cell body depolarizing the cell body. This creates a positive charge on the extracellular side of the cell body and basal dendrites. A movement of positively charged ions from the cell body and the basal dendrites to the apical dendrite generates extracellular potentials (Magee, 2000; Hallez et al., 2007; Buzsáki et al., 2012). These events create two vertically oriented dipoles of opposing polarity in pyramidal cells. This is due to the arrangement of these cells. Pyramidal cells are arranged with cell bodies in deeper laminae and dendritic arbors directed upward to the surface. Neurons must be regularly arranged so that they amplify each other's extracellular potentials. For this reason neighboring pyramidal and surface cells contribute the most to the EEG signal as their the axes of their dendrite trees are parallel to each other (Hallez et al., 2007).

The flow of current through the extracellular space and the relationship between recordings at a distance of the source is described by the volume conduction theory (Schaul, 1998; Rutkove, 2007). This refers to the to the spread and conduction of extracellular potentials through the biological tissue between the source and the sensor. This bypasses the delicate wiring of the brain but spreads according to standard laws of electrodynamics through the tissue (Plonsey and Heppner, 1967; Hallez et al., 2007). Volume conduction makes measurement of EEG possible in the first place, yet makes separation and interpretation of EEG signals difficult.

Common EEG recording techniques measure the difference of the electric potential of a surface electrode with respect to a reference surface electrode. After the charges reach the electrodes, they are transmitted through cables to a high impedance amplifier. To resolve the high frequency content of EEG, the amplified signal needs to be sampled by an analog to digital converter at a high sampling rate. The sampling rate typically ranges from 250 to 2000 Hz and must be greater than twice the Nyquist frequency to ensure an adequate sampling and to minimize aliasing. The Nyquist frequency is the highest frequency that is of interest to be detected. If the Nyquist frequency is 600 Hz, then the sampling rate should be at least 1200 Hz to avoid aliasing. Here aliasing refers to the effect of under-sampling when higher frequencies are present. This results in the creation of lower frequencies in the analog-to-digital converter (Sinclair et al., 2007). As an example, Waterstraat et al. (2012) used a sample rate of 2000 Hz while recording EEG with the purpose of investigating these frequencies around 600 Hz. After recording, the data is stored on a computer hard drive. Further signal processing and analytic processes involve the removal of uninteresting signals and noise from the raw data.

After filtering, the clean signal appears as waves that are the product of the rhythmic activity of clusters of neuronal cells. It was thought that brain rhythmicity was generated from medial thalamic structures. It is now thought that neurons in the nucleus reticular thalami are the pacemaker. These neurons discharge rhythmically to the thalamocortical relay. This leads to synchronous excitatory postsynaptic potentials (EPSPs) (Schaul, 1998). The brain's rhythmic activity is defined by its occurrence at each second, therefore frequency in Hertz (Hz).

Brain rhythms can occupy several frequencies. Here we attempt to summarize and give brief examples about brain rhythms and their functioning. The lowest frequency band is the delta (δ) band. It ranges from approximately 0.3 to 4 Hz. This band is predominant during sleep and in infant children. Its manifestation in adults is associated with learning and attention deficits (Clarke et al., 2001). The next frequency band in the spectrum is the theta (θ) band. It occupies the frequencies from 4 to 8 Hz. Theta waves are associated with repression or inhibition of behavioral activities, drowsiness and with creative or spontaneous states. Occupying the next frequency band from 8 to 13 Hz are the alpha (α) waves. These were the first observed by Hans Berger and therefore called alpha. Alpha waves occur during relaxation and closed eyes state and are associated with the inhibition of certain functions in the brain (Goldman et al., 2002). Beta (β) waves occur in the frequency range from 13 to 30 Hz. Beta activity is related with anxiety, irritability, agitation, sleep disturbances and addictions (Prichep and John, 1992). Gamma (γ) waves constitute the remaining frequency ranges from 30 to 100 Hz. This spectrum band is thought to be relevant for sensory and cognitive related brain functions. Gamma waves are thus involved in the complex activities of information processing (Colgin et al., 2009). They may also be related to motor visual processing and facial features expression (Muthukumaraswamy, 2010; Tang et al., 2011). Activity at higher frequencies are also present in the central nervous system. For example, frequencies situated around 600 Hz. These oscillations consist of a brief burst of activity, labeled often labeled as sigma-burst (σ-burst). The previous mentioned frequencies are considered to be generated by post synaptic activity. However higher frequencies, at around 600 Hz, are thought to originate from spiking activity. That is, the added-activity from single neuron cell spiking activity. Alterations in the amplitude and latency of the sigma-burst were observed under, reduced attention, general anesthesia and different stimulation paradigms (Waterstraat et al., 2012).

Specifically regarding movement, EEG activity is used as an indicator of movement initiation, prediction of its direction and even the limb that could be active during motion (Ahmadian et al., 2013). Human EEG is synchronized with muscle contraction (Salenius et al., 1996, 1997; Schoffelen et al., 2008) and is coupled with gait phase (Gwin et al., 2011). EEG rhythm changes before movement occurs for example as the Bereitschaftspotential or alpha and beta event related desynchronization (ERD). The bereitschaftspotential is a negative cortical potential which occurs around 1.5 to 1 s before the onset of a voluntary movement (Kornhuber and Deecke, 1965; Shibasaki and Hallett, 2006). ERDs are a short lasting decrease of frequency power in the alpha and beta bands that appear about 2 s before movement (Pfurtscheller and Neuper, 2003). As a practical example, these signals are used to decode a subject's movement intentions and provide control of an exoskeleton which aids the subject during locomotion (Kilicarslan et al., 2013).

### 2.1. EEG artifacts

Inherent with the measurement of brain activity are noise and artifacts. During recording, several sources of artifacts exist and therefore several kinds of noise contaminate the raw signal. The first most evident artifact, that occurs in recordings during movement are muscle activity artifacts. Muscle artifacts have their origin in the head and neck musculature which become active during head movement or stabilization during motion tasks (Gwin et al., 2011). Electromyographic (EMG) artifacts are the most difficult to deal with due to the fact that their spectrum overlaps with EEG activity, mainly with beta and gamma waves (Brown, 2000).

Other artifacts arise from sweat bridges, electrodes and cables movements, cardiac activity such as ballistocardiographic artifacts and eye movement. Sweat bridges occur when the person sweats and the salt and water form a contact bridge between two or more electrodes or simply alter the impedance of the electrodes. The electrolytes produced by the sweat glands create a battery effect causing a low frequency artifact. Eye movement and blink artifacts are also a source for EEG noise. In case of the use of a common average reference, they tend to affect the frontal electrodes causing a typical effect easy to identify in the raw data and in a topographic plot of the scalp. In the case of a nose reference they influence all electrodes. Electrode movement artifacts occur when the contact of the electrode with the scalp is disturbed, which results in a rapid change of impedance. Ballistocardiographic and cardiac activity artifacts happen when the pumped blood causes a mechanical movement on an electrode that lays on top of a blood vessel or is contaminated with heart electric activity. These are also easy to spot artifacts because they are rhythmic and with a much higher amplitude than EEG (Tyner et al., 1983). In sections, 3.5 and 4.2 we will present suggestions for the reduction of artifacts during recordings and during analysis.

## 3. Recording hardware, software, and techniques

In this section, we present hardware, software, and techniques to deal with the previously described artifact issues and the recordings of body and brain dynamics during movement, with an emphasis on spatial resolution.

### 3.1. Amplifiers, electrodes, and cap types

#### 3.1.1. Amplifiers

Over the past decades amplifiers have been optimized to improve input impedance. Today's amplifiers do not therefore alter the surface potentials. However, the surface potential is a result of the brain activity but not necessary for the brain activity itself. von Tscharner et al. (2013) has recently shown, by a model computation, that because of the relatively low inter electrode resistance, lateral currents between electrodes cause signals from neighboring electrodes to record mixed signals. Thus, signals contain information from both locations. Therefore, high impedance potential amplifiers do not allow optimal spatial resolution. The authors have shown this for EMG signals but this is most likely also the case for EEG signals. As an alternative, researchers may use trans-impedance amplifiers (electric current amplifiers). A trans-impedance amplifier removes or injects charges to keep the electrodes at ground or reference potential at all times. It yields a measurable voltage output proportional to these currents and thus to the EEG signal. von Tscharner et al. (2013) demonstrated that the trans-impedance amplifier significantly improves spatial resolution of EMG recordings because the inter-electrode cross talk is reduced. Hence, this method can perhaps improve the spatial resolution for the EEG signals.

#### 3.1.2. Electrodes

Traditionally, the most use kind of electrodes type are wet electrodes, that is, an electrode that uses an electrolyte gel, or other means, to convey the signal from the person's scalp to the electrode pin that is coated with Ag-AgCl. This coating is used to obtain a low resistivity between the skin and the electrode and the conductive gel minimizes the electrochemical contact potential. Nevertheless, these electrodes require a time consuming preparation, especially while using a high number of electrodes for source analysis studies. After the measurements, the subjects also have to wash their head to remove the conductive gel. In addition, during longer data collection sessions, the gel may dry impairing signal conductivity. This limits the study of behavior, the development of brain computer interface for every day use, long term EEG studies or measurements in extreme conditions such as in space. In order to address these issues, researchers in recent years have developed dry electrodes.

A review by Liao et al. (2012) explores several solutions for dry electrodes alternatives. Most dry electrodes are of three types: dry micro-electromechanical system sensors (MEMS), dry fabric-based sensors and hybrid dry sensors. Additionally, a technology mentioned by Liao et al. (2012), are Photrodes™. These are a NASA spinoff in collaboration with the company Srico, Inc (Sawbury Blvd Columbus, OH 43235-4579, USA). A Mach-Zehnder interferometer measures the electric activity via the electro-optic effect that modulates a light beam. Just like other dry sensors, these also do not require skin preparation (Kingsley et al., 2004). In terms of performance, Estepp et al. (2009) showed that the correlation between wet and dry electrodes ranged from 0.45 to 0.82 depending on the electrode position on the participant's head. Additionally, Grozea et al. (2011) tested bristle-sensors against wet sensors and verified that the average coherence of the bristle-sensor/gel-based pair was above 80% of the average coherence of the two employed gel-based electrodes, from 7 to 44 Hz. In addition to that, in the frequency range around 10 Hz, the average coherence between dry and wet electrodes reached 90% of the wet-wet average coherence. For dry non-contact electrodes, Chi et al. (2012) reports a correlation between dry non-contact and wet electrodes, above 0.8 for half of the participants and for dry contact electrodes, a correlations of 0.9. Chi et al. (2012) explain that the lower signal correlation seen with non-contact electrodes and contact electrodes is due to signal degradation and susceptibility to movement artifacts when using the electrodes through hair. In summation, most of these sensors performed well, however there is no single study that tested these different devices with the same condition. In addition, the MEMS may cause injury or skin irritation due to friction of the contact surfaces with the scalp skin. Thus, researchers are advised to take this into account and judge the trade-off between technologies and take into account which conditions these perform better when designing studies (Liao et al., 2012).

**Figure 1:**
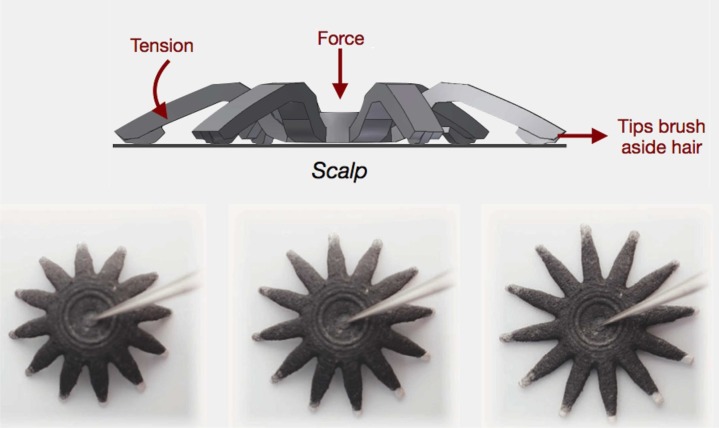
**Schematic of Cognionics active dry Flex sensor**. Top: deformation of the sensor brushing hair aside. Bottom: Top view of the sensor spreading over a surface. The sensor has 15.24 mm in diameter and 11.43 mm in height. Picture courtesy of Cognionics. (Cognionics, Inc., San Diego, CA 92121).

To address the problem of movement noise and other signal interference, it is recommended to use active electrodes and shielded cables (Metting van Rijn et al., 1990, 1996). Active electrodes amplify the signal at the source, have a high input and low output impedance thus reducing the noise created by stray potentials and cable movements (Metting van Rijn et al., 1996). Grozea et al. (2011) and Chi et al. (2012) elaborated on solutions for active, dry electrodes. One commercial product of a MEMS electrode is the g.SAHARA by g.tec medical engineering (g.tec medical engineering GmbH, Sierningstrasse 14, 4521 Schiedlberg, Austria). Cognionics (Cognionics, Inc., San Diego, CA 92121) proposes a different approach to active dry electrodes, with their Flex Sensors in Figure 1. This approach provides a solution to the hair interference problem which became evident when using other previous dry electrodes (Chi et al., 2012). The electrodes are made from a 3D printed nylon material and are provided with a set of angled appendages, similar to legs, which when under pressure deform and flatten. This brushes the hair away and increases contact with the scalp surface while reducing hair interference. When compared to dry electrodes these show a correlation of about 0.9 between the wet and dry signals (Chi et al., 2013). However these electrodes can only be used 20 to 30 times. Nonetheless, wet active and shielded electrode solutions exist, just like the actiCAP electrodes, distributed by Brain Products (Brain Products GmbH, 82205 Gilching, Germany).

#### 3.1.3. Caps

The number and spatial distribution of EEG electrodes in an EEG electrode holder cap influences the spatial resolution and accurate source localization. Junghöfer et al. (1999) and Gutberlet et al. (2009) recommend a minimum of 64 channels with equidistant positions covering the lower areas of the head to record activity from these areas of the brain. A significant number of electrodes are recommended for independent component analysis (ICA) based artifact removal methods (Michel and Brandeis, 2010). For instance, Lau et al. (2012) showed that up to 125 electrode channels improve the ICA decomposition. On the other hand, it is possible to localize the two most robust sources with only 35 electrodes (Lau et al., 2012). Therefore, the number of channels may depend on the study objectives. Higher resolution may be necessary when measuring EEG activity during motion and correlating the EEG signals to EMG signals from specific muscles. The general view that for the localization of more sources, more electrodes are required may be misleading because the inter-electrode resistivity drops with shorter inter-electrode distances and thus crosstalk among electrodes limits the spatial resolution (von Tscharner et al., 2013). Future research may therefore take advantage of combining measurements using trans-impedance amplifiers (mentioned above). However, the main limiting factor for analyzing EEG activity acquired during motion is most likely noise and movement induced artifacts. This will affect source localization. Thus the maximal appropriate number of electrodes will depend on how well one can control the mechanical influences and the inter electrode cross talk. Nonetheless, as signal acquisition and pre-processing techniques improve, one is approaching a technology that provides sufficient resolution and stability to obtain movement and behavior related information from the EEG.

**Figure 2:**
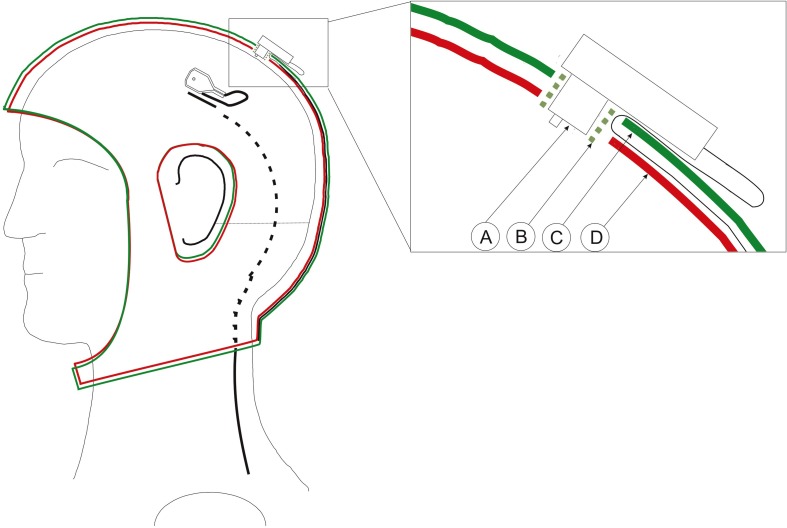
**Schematics of the adapted double layered actiCap**. Left: Cap with an electrode whose wire enters into the cap layer. The black cable is on the surface and it is depicted as a dashed line as it enters into the first layer of the cap. It leaves the cap at the bottom. Right: Close-up of a transverse view of an electrode inserted in the cap. A, Electrode; B, Plastic electrode holder; C, Upper cap layer. D, Lower cap layer. The green plastic electrode holder helps to fix both layers and the electrode. The cable passes trough the first layer to be fixed between both layers.

Double-layered caps prevent cables from moving by restraining the cables between the layers. Thus eliminating a source of artifacts. The most commonly used ones are the BrainWave cap (Medi Factory BV, Buizerdstraat 3a, 6414 VT Heerlen, The Netherlands) or the WaveGuard™ (ANT-Neuro, Colosseum 22, 7521 PT Enschede, Netherlands). Alternatively, researchers can combine two of their present caps and accommodate the wires between the layers. This works particularly well with the actiCAP from Brain Products as seen in Figure 2.

**Figure 3:**
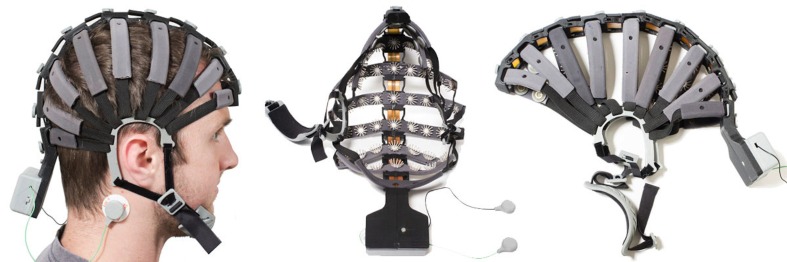
**Left: Subject wearing the headset**. Reference electrodes are allocated on the side of the neck. Middle: View from the interior part of the headset with the structure that holds the electrodes. Right: Headset maintains its shape when not utilized. Picture, courtesy of Cognionics (Cognionics, Inc., San Diego, CA 92121).

Cognionics provides a high-density dry electrode EEG headset system which supports up to 64 channels (Chi et al., 2013), illustrated in Figure 3. This system integrates the Cognionics Flex Sensors described just above, and Cognionics version of the wireless acquisition unit, described in section 3.3.2. This design is important in order to keep adequate pressure on the sensors and thus ensures contact between sensor and scalp. The headset has concealed and restrained electrode cables; eliminating cable movement and thus cable noise. Additionally, it seems to require minimal preparation and only small adjustments on pressure to ensure adequate signal collection.

### 3.2. Spatial localization of electrodes

Source localization techniques attempt to determine the generators in the brain that gave rise to a given scalp potential map. This is done by combining the EEG data with MRI images, thus providing a 3D representation of the possible cortex electric activity sources. However the accuracy of source localizations is influenced by the precision of the spatial localization of the electrodes in a 3D volume (Wang and Gotman, 2001). The information about electrode positions allows for the co-registration of the sampled EEG data with the study participant's own anatomy. (Michel et al., 2004). Three steps are necessary to obtain EEG sensors localizations: digitization of the electrode positions, electrode labeling and finally coregistration of the labeled 3D positions on the on the headmodel (Koessler et al., 2010). For more details on EEG source imaging readers can consult other studies (Grave de Peralta-Menendez and Gonzalez-Andino, 1998; Pascual-Marqui, 1999; Michel et al., 2004; Hallez et al., 2007).

Several methods exist to determine the electrode positions. The first and most described method is the 10–20 system, in which the electrode distances between adjacent electrodes are either 10 or 20% of the total front-back or right-left distance of the skull (Jasper, 1958). This system is limited, because the placement of electrodes is user dependent, therefore prone to inherit error of subjectivity. It also does not account for small inter electrode positioning differences and the subject's own anatomy. Furthermore, many of todays EEG electrode systems are implemented on elastic caps or some other kind of structure that allows a faster placement of electrodes on the head. Electrodes integrated in this kind of structure have a roughly pre-determined position, which adapts to the person's head (Michel et al., 2004).

To address these problems, researchers have several options that digitize positions of each electrode: The ELPOS system (Zebris Medical GmbH, Max-Eyth-Weg 43, D-88316 Isny, Germany) and the FastTrack system (Polhemus Inc, 40 Hercules Dr, Colchester, VT 05446, United States of America) can be used for this purpose. These systems automatically label each electrode. However, the digitalizations take about 20–40 min or more when multiple electrodes systems are employed and are user dependent, as the user must touch each electrode in order to acquire it's position. A study from Engels et al. (2010) further exposes some limitations and factors that influence the precision of systems such as FastTrack.

A less user dependent method for acquiring electrodes positions was described in the patent EP 2 561 810 A1 by Engels et al. (2011). This method uses at least 14 cameras that are arranged around the subject to determine the positions of reflective markers attached to the electrodes. The system detects and labels the electrodes automatically. However this method also needs an MRI scan of the person's head and a laser digitized scan of part of the person face and head, which is time consuming, impractical and expensive.

Russell et al. (2005) describes a photogrammetry system. This device shows reliable results and seems easy to use. A limitation may be that this system only works with a geodesic electrode array from Electrical Geodesics (Electrical Geodesics Inc., Eugene, Oregon, United States of America).

Ettl et al. (2013) demonstrate another optic system for the spatial detection of electrodes. This system is user independent, highly accurate and fast. It uses a hand-held, motion-robust, optical sensor based on Flying Triangulation (Ettl et al., 2012). The measurement occurs when a single-shot sensor acquires images yielding sparse 3D data. Afterwards, the data is aligned and the current measurement process is visualized in real time. Then, a dense 3D model of the object is obtained (Ettl et al., 2013). This system shows promise, although, it still does not detect and label electrodes automatically.

### 3.3. Wired and wireless EEG systems

Brain activity may be recorded by means of wired or wireless EEG systems. Nevertheless, study possibilities differ substantially, according to the systems' characteristics and as subjects are more restrained with a cable system than with a wireless system. Here we describe some of these systems and propose some means for allowing the recording of EEG during motion with wired systems. Additionally we review wireless systems that show promise for recording EEG during motion. Finally, we present suggestions on how to decrease motion related artifacts and suggest software for recording brain and body dynamics during movement.

#### 3.3.1. Wired EEG systems

With wired EEG systems the subject must remain constrained to a location and move only in that area. However, some solutions for the use of cable based EEG system during movement exist:

Most EEGs recorded while moving were performed using a cycle ergometer. The reason for this is that cycling does not create stepping impacts that provoke strong neck muscle contractions and electrode movements. Typical examples of studies that employed this methodology and successfully filtered the data to remove most artifacts are Brummer et al. (2011), Hilty et al. (2011), and Schneider et al. (2013). A strategy used by Jain et al. (2013) can further help with artifact reduction during cycling. Jain et al. (2013) used a recumbent cycle ergometer in an attempt to decrease neck muscle contractions, electrode movements and other motion-induced artifacts.

**Figure 4:**
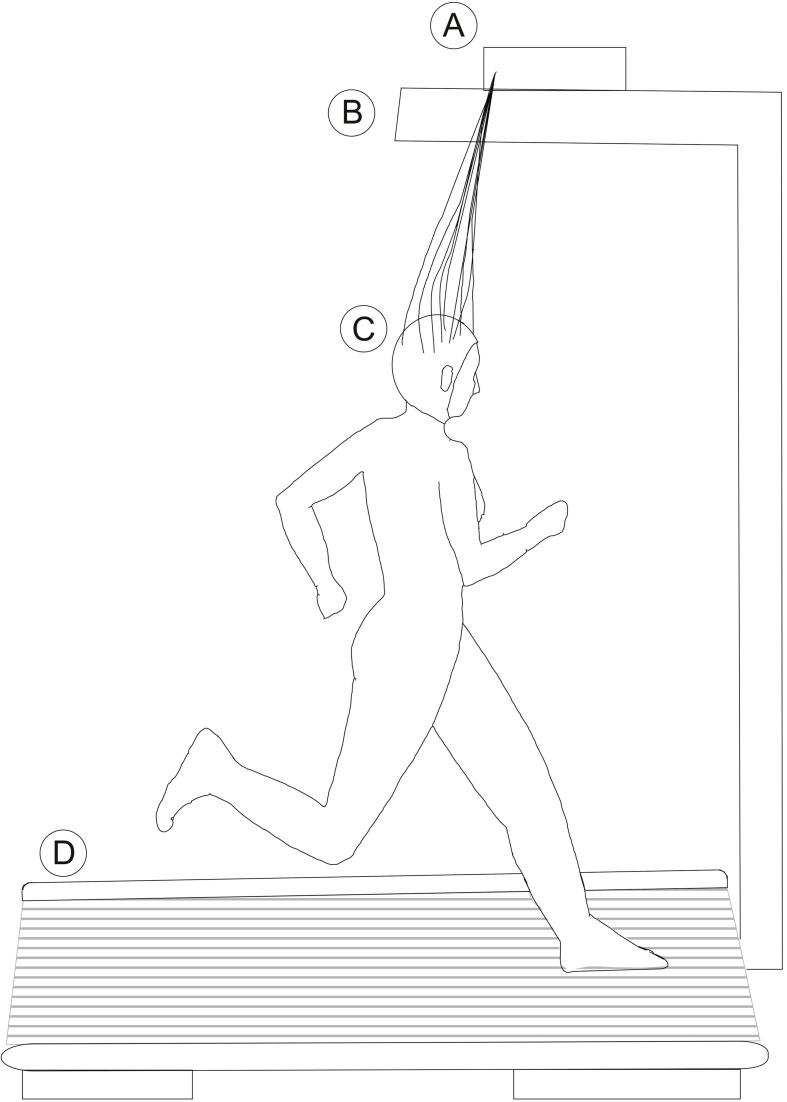
**Schematic of an over head holder for EEG amplifiers**. A, Amplifier; B, Arm holding the amplifier; C, Subject running on a treadmill (D).

For other tasks, such as running or walking, we may look at the examples of Gramann et al. (2010), Gwin et al. (2010, 2011), and De Sanctis et al. (2012). They used a customized wired EEG system that allowed the subject to run on a treadmill. The electrodes cables were attached to the amplifier mounted above the head as seen in Figure 4. However, the subject's movements were restricted due to the limited cable length. This method allows the recording of EEG during walking or running, although cable movements induce extra noise to the data (Gwin et al., 2010). This showed how important it is to restrain the cables and make use of solutions like the ones shown in section 3.1.3.

**Figure 5:**
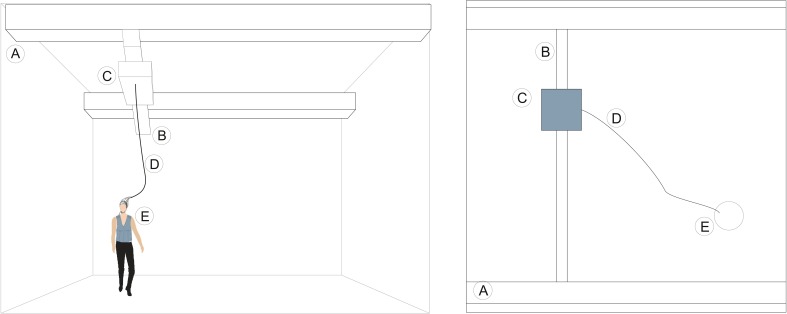
**Concept and schematics of the adapted overhead crane**. In this figure we can see the relative position of the subject and the overhead crane. A, Wall mounted lateral rails; B, Movable central beam; C, Amplifier can be moved along the central beam; D, Data cable between EEG cap and the amplifier. The subject (E) can move freely in the measurement volume that is covered by the overhead crane.

Researchers may also utilize a modified overhead crane in a large room, as shown in Figure 5. The overhead crane carries the amplifier and a pre-recording system above the subject's head, which in turn is connected by cables to the computer that records the data. This system allows the subjects to move around the designated large space. The overhead crane movements can be controlled by a feedback loop mechanism using proximity sensors, information from a MOCAP system or simply by manual control. Additionally, the overhead crane movements can be controlled by a passive system that consists of a cable attached to a body harness or vest, worn by the subject and each time the person moves, it induces it to move along.

#### 3.3.2. Mobile EEG systems

Recently, developers have optimized wireless EEG systems that facilitate mobile recordings of brain activity. These offer an advantage compared to wired systems because the person is less restricted in movement range and types. The electronics are much smaller than in the conventional devices and allow the replacement of cables that transmit the data from the EEG cap to the computer.

**Figure 6:**
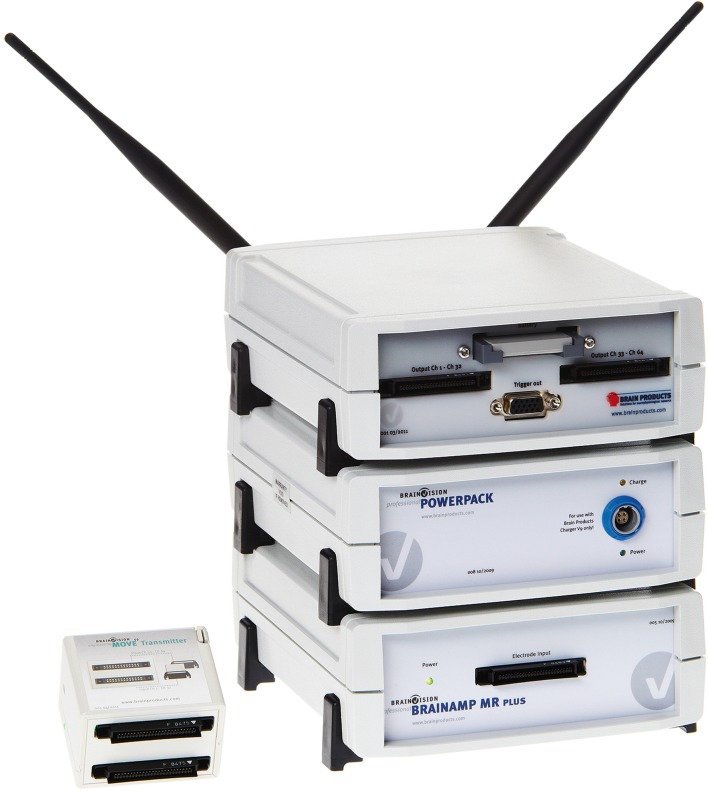
**MOVE wireless EEG acquisition system with amplifier**. Left: Transmitter, which is carried by the subject. Right, top to bottom: (1) Receiver with a small battery inserted, (2) Battery pack, (3) Amplifier. These are the static parts of the system. Picture, courtesy of Brain Products (Brain Products GmbH, 82205 Gilching, Germany).

The MOVE system, in Figure 6, replaces the cables between the electrodes system and the amplifier. After connecting the transmitter to the electrode control box, the data is transmitted via radio signals to the receiver which then sends the data to the amplifier. The transmitter pre-amplifies and digitizes the raw signals from the electrodes. The receiver then converts the signal back to an analog signal. This system can be used in addition to wet active electrodes system, such as the actiCAP from Brain Products. Moreover, the MOVE system works with several types of EEG amplifiers.

A study by Bulea et al. (2013) demonstrates the use of the wireless system MOVE. The video part of this study can be found via the link http://www.jove.com/video/50602/. In this study the subjects perform a series of exercises during data acquisition such as walking through a predetermined course in a large room, sit to stand and treadmill walking. Kilicarslan et al. (2013) used the MOVE system to acquire the brain activity of a paraplegic patient who controlled an exoskeleton with his thoughts.

Each MOVE unit can host a maximum of 64 electrodes. However, up to 5 units can be used at the same time in parallel for additional channels, or testing more than one subject at the same time. This receiver works best when it is less than 6 m distance from the transmitter. Whenever the connection is interrupted, the receiver sends a TTL marker to the amplifier and a second one when the connection is reestablished and stable. This allows the user, during the analysis phase, to know when the problem occurred. This may be a limitation, as it requires close proximity to the receiver or spatial dislocation of the receiver. All components, including the electrodes system, are powered by small long life lithium batteries, which hold the system functional for about 9 h. The manufacturer also specifies that the system has 16 bit resolution and operates at a maximal sampling rate of 954 Hz.

Another available system that allows high-density EEG recordings is the eegosports™ from ANT-Neuro. In an innovative project, much like Kilicarslan et al. (2013), researchers utilize this system to create a brain controlled exoskeleton, with the purpose of optimizing the rehabilitation of paraplegic patients. The MINDWALKER Project (Gancet et al., 2012) can be accessed under https://mindwalker-project.eu.

**Figure 7:**
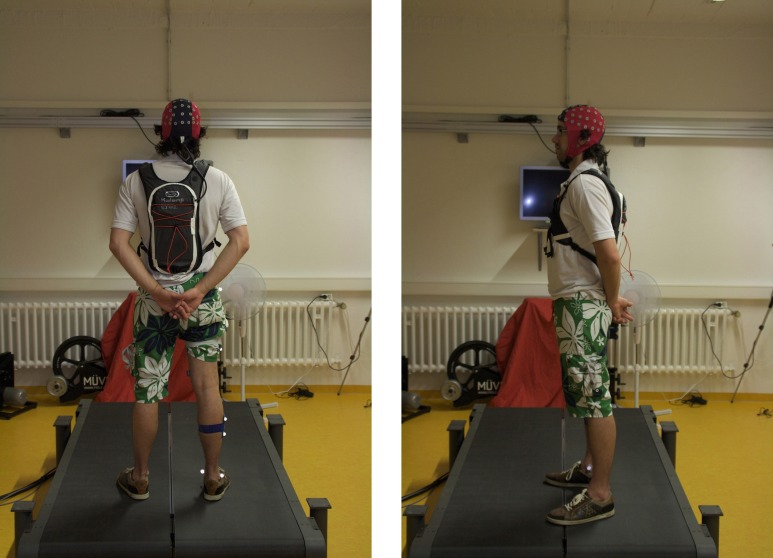
**ANT-Neuro eegosports™ wireless EEG system**. Left: Posterior view: A study participant wearing the EEGOSPORTS WaveGuard EEG cap. The cap cable goes inside the backpack where it connects to the amplifier and Ultrabook. Right: Lateral view.

The eegosports wireless system uses a different approach: it uses a small amplifier and a VAIO™ Ultrabook® (Sony Corporation, Konan, Minato-ku, Tokyo 108-0075, Japan) laptop worn in a small backpack. EEG signals enter the device at the connectors and are pre-amplified. Afterwards, they are sampled in an A/D converter located in the amplifier case. The signals are amplified and pre-recorded locally. The computer sends the data wirelessly to the remote computer where it is stored. This approach allows for the temporarily store data during unstable connections. The risk of lost data is thus minimized. The system has the maximum capacity of 64 EEG electrodes and part of these can be used as EMG bipolar electrodes. Furthermore, this system works with the ANT 64 EEG electrode array WaveGuard cap. As described in section 3.1.3, the two layers of fabric fix the electrode cables, thus potentially reducing cable movement artifacts created during motion. However, this cap utilizes passive electrodes with all disadvantages compared to active electrodes systems, even though these are shielded electrodes. Nevertheless the data obtained in a mobile setting is of sufficient quality for use in sophisticated analysis (Ehinger et al., 2014). The amplifier weights around 500 g. The whole system is light and small enough for a person to transport it (Figure 7). No cables restrict the person to any location. One issue is the temperature generated by the laptop, which may become uncomfortable and change the subject's body temperature. This increase in body temperature is undesirable as it may cause the subject to sweat. An advantage of this system is a maximum sampling rate of 2048 Hz and a resolution of 24 bit. Similarly, the eegosports is powered by integrated batteries with an operating time of up to 6 h.

**Figure 8:**
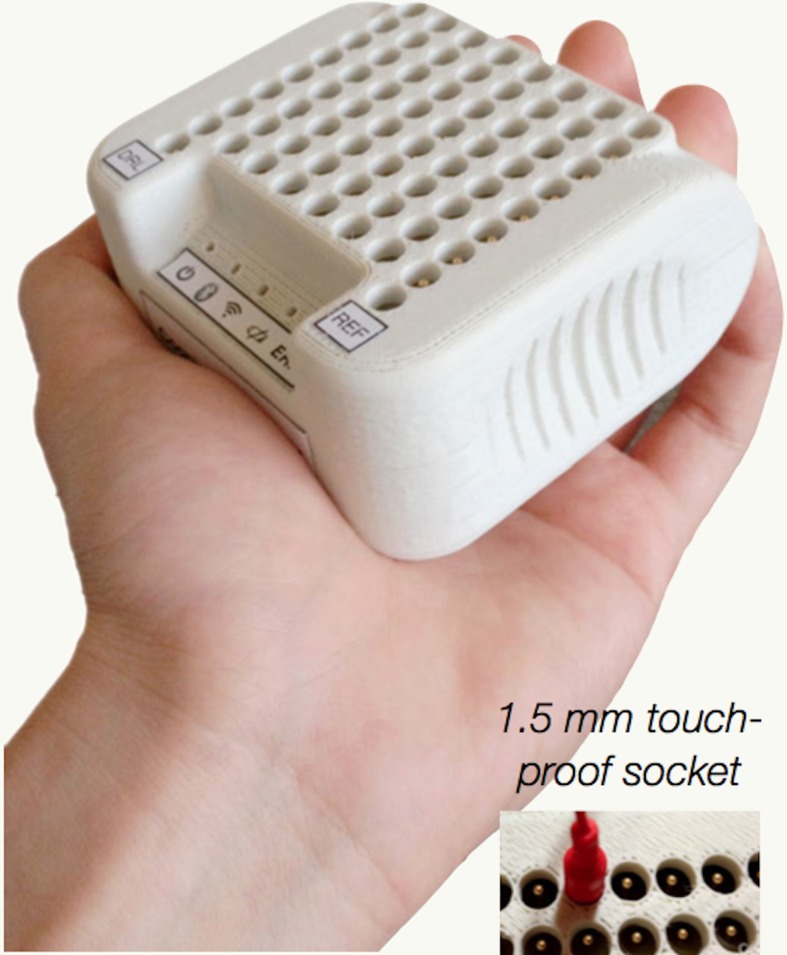
**Cognionics wireless EEG acquisition unit**. This unit holds the digitizers, amplifier, micro controller and wireless receiver. It is designed to work with standard 1.5 mm touchproof lead wires. Picture, courtesy of Cognionics (Cognionics, Inc., San Diego, CA 92121).

The last wireless system we would like to describe is the Cognionics wireless EEG acquisition unit with 64 channels with a maximum sampling rate of 300 Hz. This unit encloses the digitizers, amplifier, micro controller and wireless transmitter as shown in Figure 8. This system uses standard 1.5 mm touchproof lead wires, thus is compatible with any device that utilizes touchproof connectors. The data is wirelessly transmitted via Bluetooth within a range of about 10 m. The system is also compatible with any computer, tablet or phone supporting the Bluetooth RFCOMM/Serial Port profile. The amplifier has a built in wireless trigger receiver. Therefore, it can work with transmitters such as the ones mentioned in section 3.5.3. Two AAA ( 44.5 mm in length and 10.5 mm in diameter) batteries can feed the system for about 6 h of data streaming. Table 1 summarizes the characteristics of the described systems.

**Table 1:** **Wireless EEG systems**.

**System**	**Max sample rate (Hz)**	**Max. nr. of channels**	**Electrodes type**	**Compatible with other amplifiers**	**Pre-recording at acquisition site**	**Transmission range (m)**	**Marker when data transmission is cut**	**Subject carries**
*MOVE*	954	160 (with 5 MOVE units with 32 or 64 channels each)	Active, wet	Yes	No	6	Yes	Small transmitter
*EEGOSPORTS*	2048	64	Passive wet	No	Yes	N/A	N/A	Backpack with amplifier and Ultrabook
*Cognionics EEG acquisition unit*	300	64	Active dry	No	No	10	No	Amplifier and transmitter integrated in the headset

Other wireless systems solutions are g.tec (g.tec medical engineering GmbH, Sierningstrasse 14, 4521 Schiedlberg, Austria) and Mindo (National Chiao Tung University Brain Research Center, 1001 Ta-Hsueh Rd., Hsinchu 30010, Taiwan). It is beyond the scope of this paper to explore every system and their capabilities in detail. We exposed the main features of some systems and we advise researchers to choose the system that suits their needs best.

### 3.4. Recording body dynamics

MOCAP and Electromyography (EMG) can be recorded simultaneously and synchronously combined with EEG recordings in order to obtain body spatial and muscular dynamics, corresponding to the specific brain activities occurring in a time window (Makeig et al., 2009; Gwin et al., 2011; Bulea et al., 2013).

#### 3.4.1. Motion capture

MOCAP is the digital acquisition of movement through the use of computers. There are a few methods for the acquisition of movement:

- Mechanical means: the person wears a kind of exoskeleton and when moving, sensors detect changes in position (Calvert et al., 1982; Sharma et al., 2013).
- Electromagnetic methods: The subject wears magnetic receivers (markers), which track the location relative to an immobile magnetic transmitter (Sharma et al., 2013).
- Inertial sensor methods: Inertial sensors such as accelerometers and gyroscopes as well as magnetometers attached to a subject's body build up a body sensor network. Through a combination of the information, one can obtain joint angles and accelerations (Cooper et al., 2009; Fong and Chan, 2010; Sabatini, 2011).
- Optical methods: A person wears light reflective (passive) or emitting (active) markers (Sulivan et al., 2006; Tobon, 2010). Cameras track these markers and the system calculates their location through triangulation methods. There are also markerless methods based on computer vision (Gavrila, 1999; Poppe, 2007).

Motion related studies predominantly utilize infrared MOCAP methods because of its reliability and accuracy. Thus we explain here this method in more detail. Most MOCAP systems use reflective markers. Dedicated software combines the acquired images from different positions and by triangulation techniques it tracks the marker's positions in space. By repeating the acquisition over time, during a movement, the system is capable of describing the trajectory of an object. Systems, such as the ones provided by Vicon (Vicon Motion Systems Ltd., Oxford, United Kingdom) and systems from Qualysis (Qualisys AB, Gothenburg, Sweden) use such methodology.

The cameras' set-up is important, as at least 2 cameras must see each reflector marker to allow for triangulation. Whenever a marker is not visible by a camera, it is called an occluded marker. The addition of extra cameras may solve this problem during motion. A camera set-up of eight units is in general sufficient to capture body dynamics while walking or running. The space where the markers can be visualized by the cameras is called volume. The larger the volume, the more cameras with will be required thus allowing that 2 or more cameras can track the markers at all times (Tobon, 2010).

When recording body dynamics, the placement of the reflective markers is important for data interpretation and movement modeling. Marker positions can differ amongst manufacturers and laboratories, which can sometimes create difficulties when comparing results. C-Motion's (C-Motion, Inc., Germantown, MD, United States of America) suggestion for markers placement can be found at the following location: http://www.c-motion.com/v3dwiki/index.php?title=Marker_Set_Guidelines#cite_ref-Serge_0-0. This suggestion from C-Motion also includes a well known markers placement guideline known has the Helen Hayes markers set (Kadaba et al., 1990). In order to place the markers on a person's body, C-Motion recommends to follow palpation guidelines of skeletal landmarks according to van Sint Jan (2007).

For MOCAP of locomotion over long distances and natural environment, i.e., field tests, Ojeda et al. (2013) developed a MOCAP mobile platform. The device consists of a wheeled platform that moves along with the walking subject. The cart position must be known in order to determine the subject's position. The authors present several methods and conclude that these methods are practical to be implemented with present-day sensors that grant accuracy of better than 1% over arbitrary distances. Therefore, researchers can possibly realize full body and brain dynamics recordings in an outside environment.

#### 3.4.2. Surface electromyography

There are two kinds of electromyography (EMG): sEMG (surface EMG) and intramuscular EMG, which is an invasive technique involving needles. In this paper, we only address sEMG. In its essence sEMG is a technique that allows the evaluation of muscle activity by recording the electric activity produced by muscles. sEMG signals are the superimposed motor unit potentials (MUAPs) from several motor units. sEMG is recorded similarly to EEG, i.e., by placing an electrode in contact with the skin.

Researchers and clinicians use sEMG in applications for the non-invasive assessment of the neuromuscular structure functions. Areas of application of sEMG methods include sport science, neurophysiology and rehabilitation. From the sEMG recordings, researchers and clinicians can monitor muscle activation patterns in order to identify pathologies or evaluate therapies and sports performance (Rainoldi et al., 2004).

sEMG acquisition is performed by placing a bipolar electrode in contact with the skin above the targeted muscle of interest. The positioning of the electrodes, condition of the skin and electrode type, are important factors for adequate signal acquisition. Therefore, guidelines for EMG acquisition and EMG data analysis and reporting, were developed by the project Surface ElectroMyoGraphy for the Non-Invasive Assessment of Muscles (SENIAM) (Hermens and Freriks, 1999; Hermens et al., 2000) http://www.seniam.org and the Society of Electrophysiology and Kinesiology (ISEK) (Merletti and Di Torino, 1999) http://www.isek-online.org.

SENIAM offers guidelines for sensor types, placement and location. However, we suggest that researchers use sensor location references that best suit their experiments. Examples of other references for sensor positioning are Rainoldi et al. (2004) for lower limb muscles and Forsberg and Hellsing (1985); Schüldt et al. (1987) for electrode locations on the face, head and neck muscles.

Developments in wireless devices help reduce cable movement artifacts and increase the freedom of movement. Wireless EMG use is therefore a good choice when brain and body recordings take place in a mobile setting. EMG wireless systems offered by Noraxon (Noraxon USA Inc., Scottsdale, Arizona, USA) such as the Desktop Direct Transmission System (DTS) can hold up to 16 channels and sample at a rate up to 3000 Hz. This system utilizes small lightweight probes attached to the electrodes, pre-amplify the signal and transmit it wirelessly over a distance of up to 20 m. The DTS can also utilize other biomechanical sensors like goniometers, inclinometers, foot switches and can be combined with MOCAP.

Another wireless EMG system is the Trigno™ Wireless system (Delsys Inc. Massachusetts, USA), which uses dry EMG electrodes. The sensors include integrated triaxial accelerometers with motion artifact suppression and can be synchronized with motion capture. The Trigno Wireless supports 16 EMG channels, 48 accelerometer channels, a sampling rate of 2000 Hz and a transmission range of 40 m. Similarly to EEG systems, the literature is lacking in studies that compare EMG acquisition systems and the signal quality obtained.

#### 3.4.3. Force plates, IMUs, and eye tracking

As researchers are not only interested in investigating the kinematics but also the kinetics of a subject's movements, force plates play an essential role in biomechanics. Usually composed of a plate with integrated piezoelectric sensors or strain gauges, force plates provide information about the forces exerted on the ground and equivalently the ground reaction forces acting on the body. Inverse dynamics algorithms can then be applied to determine the forces and moments acting on the body and joints during dynamic movements such as gait, running, cutting movements etc. (Robertson et al., 2004).

As single force plates can pose problems with acquiring valid data due to bad foot placement which in turn requires a high number of trials (Oggero et al., 1998), a more and more common way to acquire kinetics during gait and running are instrumented treadmills. For measuring each foot separately during gait with double limb support phases, split-belt instrumented treadmills are used. The advantage of using instrumented treadmills is that data can be recorded continuously allowing measurements with a high number of strides in less time. Nevertheless, the gap in split-belt instrumented treadmills might affect kinematics and kinetics as the base of support is increased (Lee and Hidler, 2008; Altman et al., 2012) and familiarization is advised (Zeni and Higginson, 2010).

The measurement of forces acting directly on the body during cycling is possible through force measuring pedals that act as mobile force platforms. Strain gauges attached to a pedal spindle in a Wheatstone bridge configuration allow for measuring the tangential and normal forces in the sagittal plane (Reiser et al., 2003). Furthermore, in order to assess human sensorimotor interactions during cycling, the seat as well as grip forces and torques may be measured with sensors attached to the seat supporting rod and the stem or handle bars (Zhang et al., 2012).

Inertial measurement units (IMUs) such as accelerometers, gyroscopes, and magnetometers allow subjects to move unrestricted of a MOCAP system's measuring volume. IMUs attached to the subjects body measure its kinematics. The combination of these body sensors can then be fused to estimate joint angles (Cooper et al., 2009; Sabatini, 2011; Tadano et al., 2013). Further advantages are the low costs and the small size that makes measurements unobtrusive and implementable in realistic everyday measurements. Wireless synchronization can ensure the synchronization with other hardware components mentioned before. When not using internal storage on data storage devices, the quality of wireless communication links must be ensured to guarantee transmission to a data recording station (Hanson et al., 2009). Specialized calibration procedures or other reference systems are required for the correct alignment of the sensors to the body and angle estimation (Favre et al., 2009). The gold standard for measuring joint angles, especially during highly dynamic movement is therefore still a marker based MOCAP system.

Because there is a close relationship between vision and movement control, the synchronous analysis of gaze and motion plays an important role in current research (Ketcham et al., 2006; Heinen et al., 2012; Causer et al., 2013). Recently, Essig et al. (2012) presented a modular approach to combine infrared MOCAP systems and mobile eye trackers for the analysis of the 3D gaze vector within the 3D MOCAP volume, while traditional eye trackers relate the gaze only to 2D video positions. One step calibration procedures can ensure the coherence between the gaze direction and the MOCAP system. The integral approach allows studying gaze during dynamic movement tasks whereas traditional studies were usually carried out under artificial laboratory conditions. Researchers are thus able to investigate perception, attention and eye-body-environmental interaction in realistic 3D environments and during realistic tasks in a integral approach.

The libGaze library presents an open-source framework to combine eye tracking with MOCAP systems for real-time tracking of gaze and the observer's positions (Herholz et al., 2008). As commercial solution, the Vicon MOCAP system and the Ergoneers Dikablis Eye Tracking Solution represent a closed approach in Vicon Nexus analysis software to track the body's position and the 3D gaze vector. Version 2.9 of the Qualisys Oqus camera system also supports the Ergoneers Dikablis eye and 3D gaze vector tracking.

### 3.5. Data recording

#### 3.5.1. Reducing artifacts during data recording

In order to deal with artifacts mentioned in section 2.1 during data recording, we present some recommendations.

**Figure 9:**
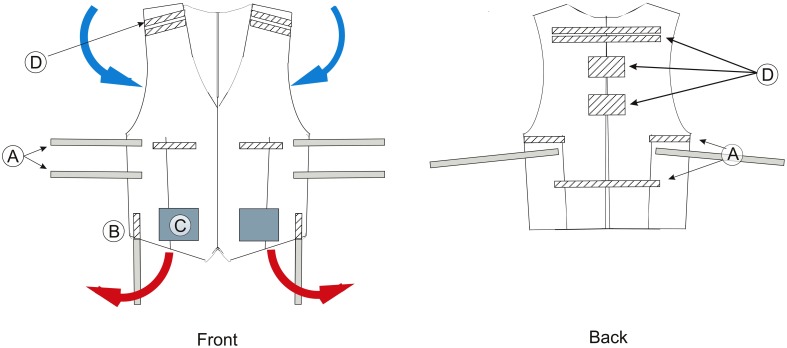
**Schematics of the modified cooling vest**. Left: Front of the vest. Right: Back of the vest. A, Hook and loop strap bands for vest size adjustment and fitting; B, Strap bands for cable holding; C, Integrated cooling unit; D, Strap bands for holding EEG equipment, such as electrode boxes or transmitters. Blue arrows indicate the flow of cool air which enter the vest. Red arrows indicate the flow of hot air, which leave the vest.

To deal with salt and sweat bridges short exercise tasks with resting intervals in an air conditioned room are recommended. To further maintain body temperature, subjects can wear a cooling ventilation vest during exercise (Pohr and Vogler, 2007). A modified version of this vest can accommodate parts of the EEG system as depicted in Figure 9. The vest opens completely and is only attached to itself in the middle section. A study by Barwood et al. (2009) shows that subjects wearing a cooling vest exercised for 18% longer time, required less rest and maintained a skin temperature lower than in control subjects. Thus, a ventilator vest can perhaps compensate for the increased heat, created by wearing EEG equipment during motion, and improve subject's performance.

To avoid electric artifacts, the recording area should be free of sources of electric interference like engines or radiation emitting devices. Mains hum create an electrical artifact at 50 or 60 Hz frequencies, for Europe and USA respectively. Notch filters can reject this artifact during post recording analysis. Further, ensuring a qualitatively good connection and online impedance check are essential in order to obtain a good signal. Finally, cables active shielding implementation help to reduce electrical noise. Solutions presented in section 3.1 reduce electrodes and cable movements. As mentioned previously, the use of a doubled layered cap effectively holds the electrodes and cables, thus minimizing this kind of artifact. In addition, researchers can use low impedance output active electrodes that pre-amplify the signal at scalp level.

Muscle activity creates a major source of noise during recordings. EMG has amplitudes from 100 to 1000 μV at frequencies from about 5 to 450 Hz. Brain activity also occupies this frequency range, raging from 0 to 100 μV. EMG artifacts dominant amplitude is in the 50 to 150 Hz band while about 90% of the EEG spectral power is present in the 1 to 30 Hz band. Therefore, when muscle activity is present, it affects much of the EEG (Shackman et al., 2009).

Due to these aspects, EMG artifacts reduction is conducted during the signal pre-processing phase by computational methods, for instance ICA (Bell and Sejnowski, 1995; Makeig et al., 1996). In this category eye blinks, cardiac and other artifacts of electromyogenic nature are also reduced during the pre-processing phase. However, adequate signal acquisition is required for better results when using ICA methods. In section 4.2 we describe some computational methods for dealing with these artifacts and reducing their presence in the recorded data. Other suggestions during recording to avoid muscle artifacts include the instruction and training of the participants to swallow and eye blink during the intervals of short recordings and avoid severe face and head muscle contractions during exercise such as weight lifting.

#### 3.5.2. Data acquisition settings recommendations

The data acquisition settings are an important step in the study design. Adequate data sampling allows successful artifact reduction using ICA methods and provides better results. Also for body dynamics recording, adequate sampling rates and the numbers of samples are necessary, depending on the hardware and analysis methods.

In infrared MOCAP, an adequate sample rate is required to allow to capture movements. For running a sample rate between 120 and 250 fps should be sufficient. For instance, Gwin et al. (2010, 2011) used a sample rate of 120 fps for running speed of 1.9 m\s. De Sanctis et al. (2012) utilized a capture rate of 100 fps for a speed of 1.39 m\s. For faster movements though, such as throwing or hitting, an increased sampling rate might be required.

For EMG sampling, a minimum of 1000 Hz sample rate is recommended by SENIAM and ISEK. This is based on the signal ranges since the significant EMG activity happens between 5 and 450 Hz. We also advise the use of standard consensual EMG sensor locations and follow recommendations of the SENIAM or ISEK. These may perhaps not be the ideal for every muscle group but it offers a base of comparison for researchers between studies. This way studies are easier to be compared (Viitasalo and Komi, 1977; Komi and Tesch, 1979; De Luca, 1997; Merletti and Di Torino, 1999; van Boxtel, 2001).

When using ICA based methods for EMG artifact removal, it is necessary to acquire enough data for the algorithms to work adequately. With Adaptive Mixture of Independent Component Analyzers (AMICA), 10,000 muscle samples may be enough find to the muscle components (Palmer et al., 2011; Delorme et al., 2012). Also when using ICA based methods, it is debatable how many data samples are necessary to find the different components. The EEGLAB FAQs web page http://sccn.ucsd.edu/~scott/tutorial/questions.html recommends to use at least the square of the number of channels. In a paper from Makeig et al. (1999) the authors used over six times the number of necessary input points and ICA, which allowed for the identification of three spatially fixed, temporally independent, behaviorally relevant, and physiologically plausible components.

Furthermore, simultaneous direct acquisition of EMG signal from the neck muscles that induce most artifact during movement (Gramann et al., 2010), can help detecting noise components and their subsequent exclusion. Thus, researchers can place EMG or EEG electrodes below the nuchal line and above the C7 process to measure activity of the muscles that provide stability to the head during motion. Also, sternocleidomaistoideus muscles perform an important role in head stabilization. Due to this stabilization function, these muscles activity, can induce artifacts. Hence, their EMG should be recorded in order to facilitate artifact removal. Researchers can consult Forsberg and Hellsing (1985), Schüldt et al. (1987), and Leutheuser et al. (2013) for suggestions of locations for these electrodes.

Subject's safety is important when performing recordings of exercises. The American College of Sports Medicine (ACSM) provides guidelines for exercise testing and prescription (Pescatello et al., 2013). These guidelines give indications to clinicians and scientists on how to perform exercise testing in healthy and unhealthy subjects and termination criteria based on physical and physiological signs. Lastly, the Borg scale of rates of perceived exertion provide a measurement tool to monitor the participant's performance and fatigue during exercise testing (Löllgen, 2004). The Borg scale measurements are correlated with oxygen consumption and heart rate.

#### 3.5.3. Brain and body data acquisitions synchronization

Synchronization of the measurement devices in the millisecond range is necessary when investigating different modalities (motion capture, force plates, EEG, EMG, etc.) simultaneously during movement. Even slight time shifts between the single devices potentially lead to a misinterpretation of the obtained results. Synchronization is also important for real-time analysis, since time shifts have immediate effects. The data from the acquisition devices are usually asynchronous due to different internal clocks, sampling rates, network and operating system delays (Delorme et al., 2011). Delorme et al. (2011) proposed a software approach for data streaming management with near real-time synchronization capabilities.

This software approach has evolved into the open-source project known as the lab streaming layer (LSL). This is a data acquisition system developed by Christian Klothe from the Swartz Center for Computational Neuroscience, Institute for Neural Computation, University of California San Diego, USA. LSL allows the exchange of time series between devices, programs and computers. It's a system for the unified collection of measurement time series in research experiments. It consists of a core transport library and a series of tools. These tools include a recording program, online viewers, importers and acquisition software. These acquisition programs can acquire data from various hardware including EEG, eye tracking, motion capture, force plates, etc., from several manufacturers.

The built-in time synchronization in LSL relies on clock offset measurement and a timestamp for each sample which are collected alongside with each actual sample data. The recording program included with LSL, the LabRecorder, collects the information, including time stamps and clock offsets, for every stream and stores it. Interested readers can consult the LSL google code page at https://code.google.com/p/labstreaminglayer/ for details, downloads and related documentation.

Another way to synchronize several devices is to use hardware synchronization. This is usually achieved trough use of TTL (transistor-transistor logic) signals, via coaxial trigger cables with BNC connectors between the devices. This eliminates potential software synchronization delays. There are several possible hardware synchronization implementations:

- To start the measurement, the system can use an initial synchronization pulse by an external trigger. Possible trigger devices are push buttons, optical systems such as photo sensors or other sensors that sense an initial movement. This method is only feasible for short measurements since a possible time drift due to the different internal clock's accuracies may lead to cumulative desynchronization between the devices with increasing measurement time.
- In order to avoid desynchronization, continuous synchronization at a fixed frame rate can be implemented. This method requires a master timebase, which regularly sends out synchronization pulses to the attached devices. Nowadays camera systems such as Qualisys Oqus system provide either external frequency outputs or locking into external synchronization input pulse sequences (Maidhof et al., 2013).
- Additionally, researchers can use wireless synchronization. The custom-built system proposed by Kugler et al. (2012) for the synchronization of wearable sensors with external devices is also feasible for the synchronization of devices such as mobile EEG with MOCAP cameras. Alternatively, researchers can use commercial systems, such as the Cognionics wireless triggering system http://cognionics.com/index.php/products/trigger.

## 4. Data analysis software and artifact removal techniques

### 4.1. Software for data analysis and visualization

**Figure 10:**
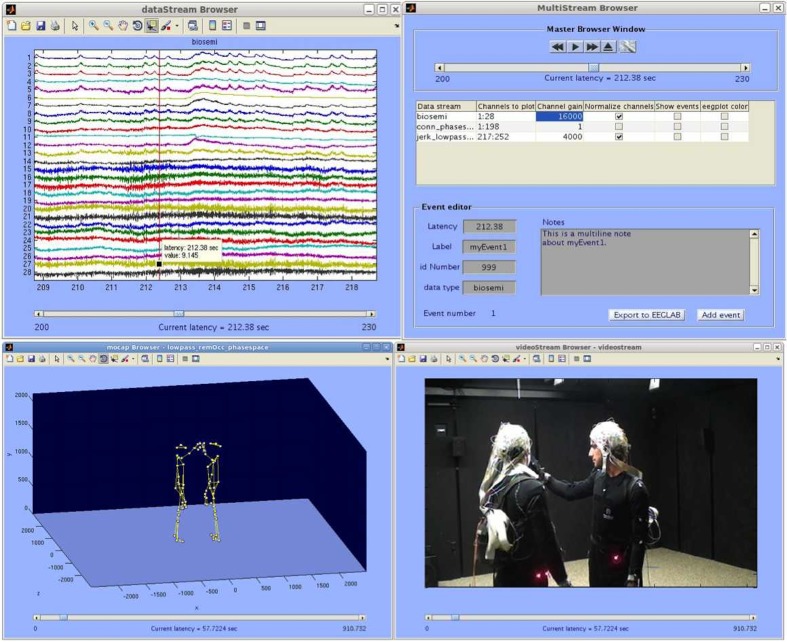
**MoBILAB multi-stream browser screen shot**. Upper Left: Data stream browser (EEG, EMG, etc.). Upper right: Master browser window. Lower left: Motion capture browser. Lower right: Video stream browser. Picture, courtesy of the SCCN and Alejandro Ojeda. http://sccn.ucsd.edu/wiki/MoBI_Lab.

To visualize and analyze synchronously captured data some options exist. Data acquired with the LSL software can be read by the MoBILAB software package. This software contributes to the Mobile Brain/Body imaging (MoBI) concepts put forward by Makeig et al. (2009). MoBILAB is designed by Alejandro Ojeda, also from the SCCN, with Nima Bigdley Shamlo and Christian Kothe. Now, this package runs as a standalone, open source, cross platform toolbox for Matlab (The MathWorks, Inc., Natick, Massachusetts, USA). MoBILAB supports the analysis and visualization of synchronously recorded EEG data, motion capture, EMG data and environmental data as seen in Figure 10.

In the issue of this same paper, Alejandro Ojeda dedicates an article to the MoBILAB software. Therefore, it is irrelevant to further detail this software here. For details, readers are invited to consult Ojeda et al. (2014) and the wiki page http://sccn.ucsd.edu/wiki/Mobilabsoftware.

A further possibility is a subsequent usage of biomechanical analysis and signal analysis software, such as Visual3D™ (C-Motion, Inc., Germantown, MD, United States of America) and EEGLab (Delorme and Makeig, 2004) or other signal analysis software. Visual3D is a product for 3D MOCAP data analysis and biomechanical modeling. It provides signal processing and biomechanical analysis tools such as 6° of freedom modeling, inverse kinematics and dynamics and can thus determine the joint angles, powers, moments, forces, velocities and accelerations during motion. Additionally, time series segmentation can be conducted with Visual3D, for example for gait cycle segmentation or any other movements using event detection based on minimum/maximum search, thresholding or template comparison on any calculated biomechanical parameter. When exported to EEGLab, the segmentation time stamps can be of further use, provided synchronized measurements, brain and muscle activity can thus be directly linked to the corresponding movements. In EEGLab, the user can then proceed with the necessary EEG signal analysis such as source localization for the specific movement task.

### 4.2. Artifact removal methods

**Figure 11:**
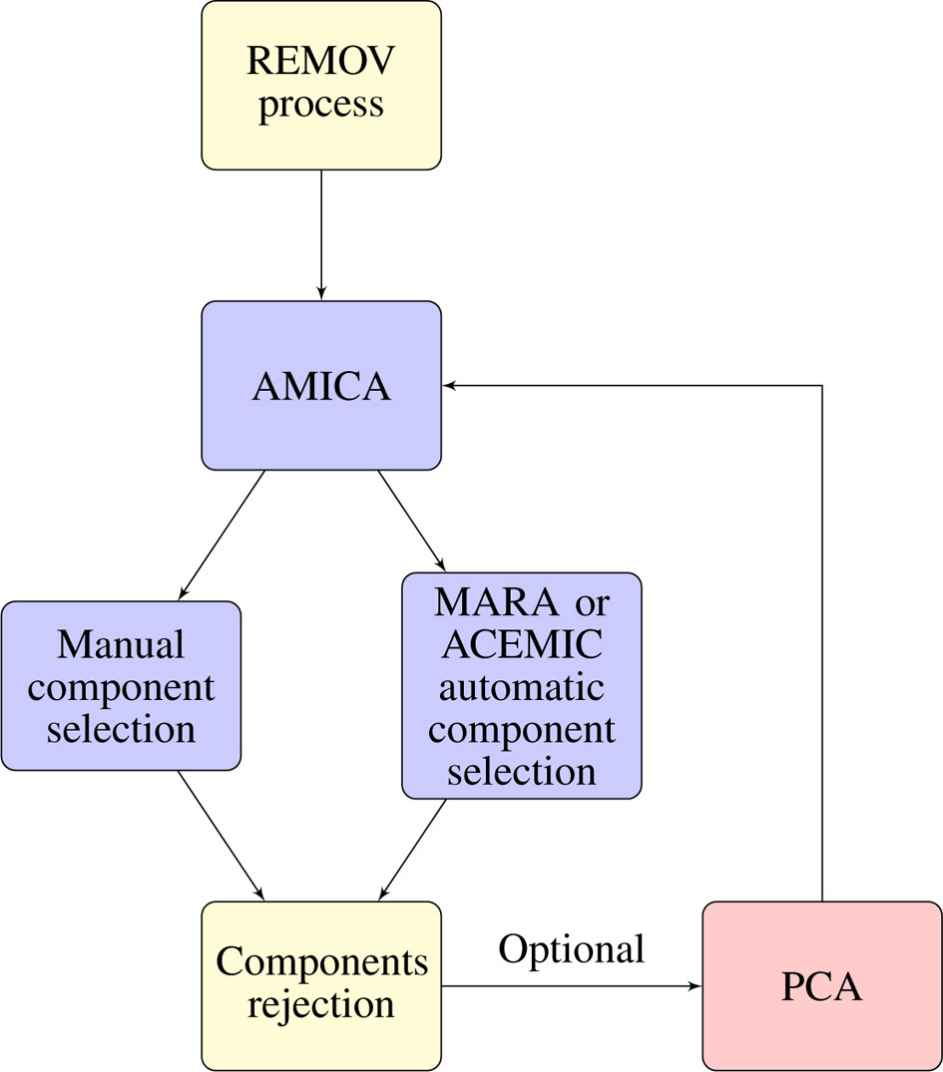
**Artifact removal process**. The first node corresponds to the *REMOV* process as in Artoni et al. (2012). The second node corresponds to the data decomposition using AMICA which was used successfully for removing movement related artifacts by Gramann et al. (2010); Gwin et al. (2010, 2011); Leutheuser et al. (2013). AMICA is a ICA method and for EEG data decomposition instructions exist at http://sccn.ucsd.edu/wiki/Chapter_09:_Decomposing_Data_Using_ICA. After signal decomposition components must be selected. Users can opt by *manual selection* or *automatic selection*. Automatic selection with MARA (Winkler et al., 2011), http://www.user.tu-berlin.de/irene.winkler/artifacts/ or ACEMIC (Gabsteiger et al., 2013) available at http://www5.cs.fau.de/research/areas/digital-sports/automatic-classification-of-electromyogenic-ica-components/. Criteria for the manual selection of EMG and other noise components are described in Goncharova et al. (2003); McMenamin et al. (2010). The next step is *component rejection*. Components can be plotted and rejected for example, using EEGLAB. Optionally, users can perform signal decomposition once again. If so, as suggested by the EEGLAB manual for ICA decomposition, to run ICA once again the data dimensions need to be reduced to the number of remaining components. Thus, users should run PCA, as instructed. After run AMICA once more and proceed again with the previously described steps. We advise, running AMICA once, remove the 4–6 (dependent of number of channels) most noisy components, running AMICA again and removing again noise components.

Signal artifact reduction procedures combine various approaches and routines to EEG artifact detection and removal. Overall, artifact removal procedures can be divided into basic and advanced processes. The basic stage of artifact removal focuses on environmentally induced artifacts such as cable noise, power line noise and impedance increase. These can be removed mostly by band and notch filters. The advanced stage involves the removal of EMG and other artifacts through methods such as ICA (Bell and Sejnowski, 1995; Makeig et al., 1996). Here we suggest a compilation of several artifact removal procedures. Figure 11 describes the complete procedure.

We suggest the REMOV process as the first stage of data cleaning thoroughly described in Artoni et al. (2012). In this step, most of the environmental artifacts are removed through filtering and noise segments rejection using BCILAB tools (Kothe and Makeig, 2013) available for download at http://sccn.ucsd.edu/wiki/BCILAB. Application of band pass filters is the inclusion of frequencies of interest and exclusion of other less interesting frequencies and noise. The REMOV procedure includes the removal of eye blinks but not the removal of EMG, heart and loose electrodes artifacts. The combination of the REMOV process with other procedures allows further reduction of artifacts.

For the removal of the remaining artifacts (heart beat, loose electrodes, ocular movements, muscular activity), researchers can use ICA methods and EEGLAB compatible tools for further processing. As of today, there exists several variations of ICA algorithms. We advise the use of the Adaptive Mixture of Independent Component Analyzers (AMICA) (Palmer et al., 2011) as it outperforms other algorithms in decomposing data (Delorme et al., 2012) and at removing EMG artifacts (Leutheuser et al., 2013). Also Gramann et al. (2010); Gwin et al. (2010, 2011) used AMICA successfully to remove walking and running artifacts from EEG data. AMICA source code is available at http://sccn.ucsd.edu/~jason/amicaweb.html. After the data is decomposed by ICA, noise inducing components must be selected. For the selection of ICA components, researchers can choose an automatic or manual approach.

Due to the typical problem of the subjective and time consuming selection of ICA components to exclude some researchers created automatic component selection tools in an attempt to reduce the user dependent factor. An is the Multiple Artifact Rejection Algorithm (MARA) (Winkler et al., 2011), http://www.user.tu-berlin.de/irene.winkler/artifacts/. This is a universal classifier of ICA components from EEG data. MARA can be used as a plugin for EEGLAB. It is based on linear methods and can be utilized with different electrode placements. This classifier was trained by experts on large data during static and dynamic situations. This algorithm identifies components from muscle, eye and electrode movements. This is an attempt to automatize the time-consuming component selection process. However, we do not know of any walking, running or sport related study that used MARA. Therefore, its performance is somehow uncertain with other movements than the one which the classifier was trained with.

Thus, Gabsteiger et al. (2013) trained a classifier for the selection of muscle activity independent components. It is designed to cover a diverse selection of exercises that stimulate the musculature that most interfere in EEG recordings during movement: the Automatic Classification of Electromyogenic ICA Components (ACEMIC). This selection of exercises should produce similar artifact patterns as seen in most exercises or movements. Evaluation of this classifier shows a 93% sensitivity and 96% specificity. ACEMIC is implemented as a plugin for EEGLab and can be downloaded from http://www5.cs.fau.de/research/areas/digital-sports/automatic-classification-of-electromyogenic-ica-components/.

Users may opt for manual selection of ICA components. For this purpose, we suggest users follow indications for data decomposition of the EEGLAB manual http://sccn.ucsd.edu/wiki/Chapter09:DecomposingDataUsingICA. EMG and other artifact component selection directions, according to their spectral and topographical characteristics, are given in Goncharova et al. (2003) and McMenamin et al. (2010). Components that exhibit high spectral power and that are located at the electrodes of the periphery, are more likely to be myogenic activity. Also, the shape of the dipole patters has to be considered. EEG activity patterns are more likely to show smooth well-localized and defined patterns. With these propositions, researchers will more accurately identify noise components that should be removed.

**Figure 12:**
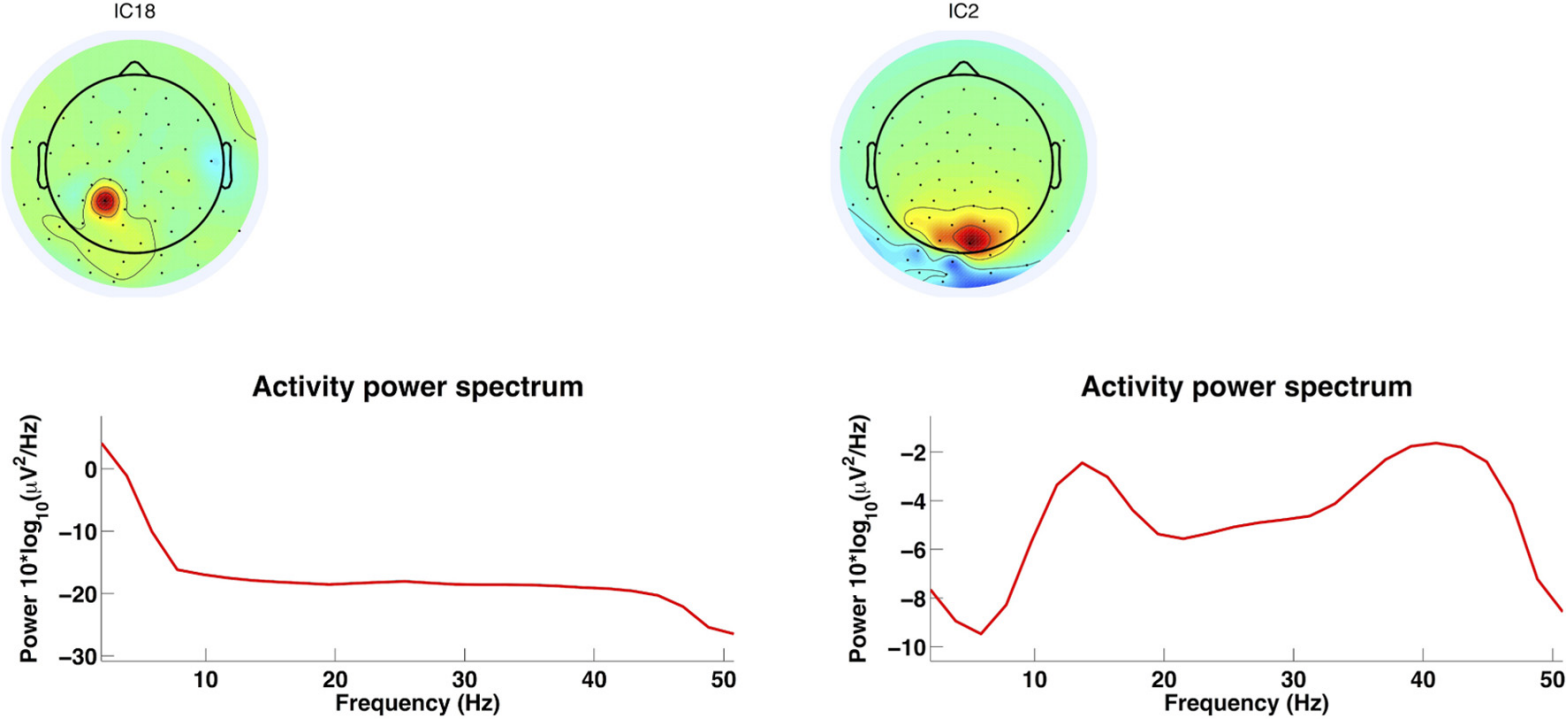
**AMICA decomposition components**. This figure shows two components from an AMICA decomposition using EEGLAB for the plots. Left: Centrally localized component shows higher power content in lower frequencies and a drastic reduction at frequencies above 30 Hz. Right: Component localized at the back of the head with high power content above 30 Hz which is consistent with EMG activity (Goncharova et al., 2003). This component should be considered for rejection which can be realized with EEGLab.

It is important to remove artifact components to keep hold of neuronal signals. Thus, Figure 12 gives an example of an EMG component and an EEG component. The selected components according to the criteria from the mentioned studies. The more centrally localized component shows higher power in the lower frequencies and a drastic reduction in power at frequencies above 30 Hz, which is consistent with brain activity components. The posteriori localized component at the back of the head at the neck has power above 30 Hz which is higher than usual for artifact free EEG. This is consistent with EMG activity and should therefore be rejected (Goncharova et al., 2003). The rejection of the components can be realized with EEGLAB as well. Further, artifact reduction techniques can be tested for overcorrection of the EEG signals. Gwin et al. (2010) did so by computing the power spectral density of the resulting signals and compared spectral power in the 1.5- to 8.5-Hz frequency band before and after application of AMICA as an artifact removal tool. There was no sign of removal of EEG signal. The artifact cleaned recordings were also tested for whether in the movement conditions it would be possible to identify a ERP time-locked to visual target (oddball) stimulus. These were nearly identical to ERPs in the baseline condition (standing). For the running condition the ERP was only visible after artifact reduction. Therefore with this methodology it is possible to remove artifacts during running so that ERPs are identifiable similarly to a baseline condition.

Another interesting and valuable approach is demonstrated by Plöchl et al. (2012). This study attempted to remove eye movement artifacts by simultaneously recording eye movements and EEG during a guided eye movement paradigm. It resulted in the creation of an algorithm, which uses eye movement information to identify eye movement related ICA-components in an automatically. Removing the detected ICs from the data resulted in the suppression of ocular artifacts including microsaccadic spike potentials, while the EEG signal remained unaffected Plöchl et al. (2012). Ultimately, this study is an example of how recording body dynamics simultaneously to EEG, can help to reduce movement induced artifacts.

Similar to ICA, Canonical correlation analysis (CCA) is also a blind source separation (BSS) method that can reduce the influence of EMG artifacts on EEG data (De Clercq et al., 2005, 2006). BSS-CCA assumes that the autocorrelation of sources that are mostly influenced by electromyogenic activity are significantly lower then the autocorrelation of brain sources. The user therefore only has to decide how many sources, i.e., components, to reject but not which ones. The toolbox is available for download at: http://www.neurology-kuleuven.be/?id=210. The BCILAB toolbox (Kothe and Makeig, 2013) includes different filters to remove artifacts. The “clean peaks” filter projects events with abnormally high power, e.g., EMG artifacts, out of the data.

## 5. Summary and conclusions

In this paper, we demonstrated methods and equipment that exist today which allow the recordings of body and brain activity during motion. Hardware, software and techniques were covered. These methodologies open a wide range of research opportunities into the cognition, motion, environment interaction and therefore, behavior fields. In fact, recording and analyzing EEG during motion remains a challenge and we hope that this paper can help researchers who attempt to dwell in this field. It is also an intention of this paper, to compile and give structure to the amounts of new methods that emerged to offer solutions for measuring and analyzing EEG and body dynamics during motion. We also speculated about future technologies such as using current amplifiers (trans-impedance amplifiers) that may allow measuring EEG with higher spatial resolution. We focused on high-density EEG and body dynamics, not addressing the field of brain computer interfaces. In future studies it will be necessary to compare different methods and hardware more often, for instance, studies comparing the reliability of different electrodes and of the recorded signal quality. If a higher spatial resolution can be obtained then it is necessary to measure more accurately and report the spatial localization of the electrodes. Generally, today's methods have reached a point where one can consider measuring EEG, EMG, kinematics, and kinetics simultaneously during motion. Thus, they open new possibilities in the field of behavior and neuroscience.

## Fundings

This work was supported by the Bayerisches Forschungsstiftung (Bavarian Research Foundation).

### Conflict of interest statement

The authors declare that the research was conducted in the absence of any commercial or financial relationships that could be construed as a potential conflict of interest.
